# Support, care and peer support for gay and bi men engaging in chemsex

**DOI:** 10.1111/hsc.14081

**Published:** 2022-10-18

**Authors:** Maurice Nagington, Sydney King

**Affiliations:** ^1^ University of Manchester Manchester UK

## Abstract

The objective of this research was to explore how gay men use drugs in their sex lives, colloquially called “chemsex”. This paper reports on a sub‐theme within the research about support, care and peer support. Longitudinal interviews were conducted with 20 gay and bi men between April 2017 and July 2019. Participants were recruited via geolocated dating apps (*n* = 17) and snowball sampling (*n* = 3). The main findings of this research are that medicalised forms of support for gay and bi men engaging in chemsex are often tardy in their responses to need, and whilst helpful for cessation of drug use, fail to address the holistic needs of the participants. A wide variety of peer support was practiced amongst the sample which often echoed previous forms of peer support practiced in the LGBT+ community. It was offered by both people who engaged in chemsex and those who did not and was highly beneficial to people who experienced problems with chemsex. However, peer support was also limited by factors such as shame and the instability of those offering support. In conclusion, we suggest that medicalised forms of chemsex support could benefit from more rigorous and rapid forms of assessment for problematic chemsex, and also provide infrastructure and training to peer support initiatives. We also suggest that medical services could learn from patients and their peers about what support needs remain unaddressed by professional services, and engage in collaborative approaches to practice development.

## What is known about this topic and what this paper adds?

- Professional support for chemsex, whilst useful for cessation of drug use, often fails to provide holistic forms of support such as helping people regain control over their sex lives.
- Peer support for chemsex is valuable, can often be provided by people without experience of chemsex, but can at times be difficult to maintain in the longer term.
- Further work is needed to examine how peers themselves can be supported, and also how they can inform service provision by highlighting unmet needs.

## 1 INTRODUCTION

### 1.1 The ideologies of chemsex

Chemsex is the combining of drugs with sex, and since 2013 it has been constructed as a public health concern, initially in London but also internationally (Bourne et al., 2014; Stuart, 2013). Stuart (2014, 2016) suggests chemsex is motivated by internalised homophobia which then drives the HIV pandemic. Yet such explanations fail to heed the caution from Malyon (1982) to avoid using internalised homophobia as a universal explanation for gay male behaviours; and, ignores other structural factors such as the poor implementation of sexual health (Tomkins et al., 2018) and PrEP services (Nagington & Sandset, 2020).

Møller and Hakim (2021) argue that research into chemsex must be contextualised within historical, social and political narratives about embodiment, sexuality and pleasure. For example, Hakim suggests losing gay venues in London secondary to gentrification leads to sexual and drug‐taking habits changing so that sex parties are organised in private venues. Alternatively, Møller (2020) explores how chemsex has integrated video conferencing centred around exhibitionist or voyeuristic approaches to drug taking, sexualised conversations and communal pornography viewing. These examples demonstrate chemsex does not have a singular cause, or expression, and instead is contingent on broader socio‐political narratives. Finally, sexualised drug use is not limited to gay men, it is a common practice across all genders and sexualities (Moyle et al., 2020). However, Kagan (2018) suggests the focus on gay men results from a “recrisising” of the “heightened feelings and spectacular images associated with the AIDS crisis sex panic in aid of newer forms of neoliberal population management” (p. 131). The polarised perspectives on what chemsex is, a pathology and public health concern defined by medical discourses versus a pleasurable sub‐cultural practice as defined by cultural theorists, are both steeped in ideology. This article does not aim to resolve these, but they do provide an important basis for contextualising various forms of support.

### 1.2 Chemsex care and support

To date, research has largely addressed chemsex as a pathology that a professionalised service treats (Montcrieff, 2018) with a clear binary between the healthy and unhealthy (Stuart, 2016) where services are evaluated in relation to political ideologies aligned with drug prohibition (Platteau 2020) rather than community‐based harm reduction needs and aims (Pires et al., 2022). These medicalised and at times coercive approaches are redolent of pastoral governance (Davis, 2019) approach to chemsex that encourages individuals to internalise drug prohibition policies, rather than engaging in nuanced peer‐to‐peer conversations that recognise the sub‐cultural complexities and pleasures of drug use. Only one paper to date has explored grass‐roots approaches to peer support, highlighting the need for culturally adapted interventions that look beyond the current constructions of chemsex experiences and support services (Pires et al., 2022). Despite this, research into chemsex services highlights that service user involvement is key to their development (Pakianathan et al., 2016) and putting in place culturally appropriate harm reduction services (Stardust et al., 2018).

This paper critiques the affordances and constraints of professionalised and medicalised forms of chemsex support, whilst also exploring how peer support can inform but not be led by pastoral governance approaches. Reflections will be drawn from how the LGBT+ community engaged in similar processes in relation to HIV/AIDS, and conclusions will explore how professional service providers can develop culturally appropriate forms of peer support.

## 2 METHODS

The aim of this study was to engage in a broad exploration of chemsex culture. The findings present a sub‐set of interviewee data which addresses the theme of support, care and peer support. Chemsex can touch on sensitive issues such as sexual assault and mental health difficulties. Therefore, longitudinal in‐depth interviews were chosen as a proven design for sensitive topics (Miller, 2017; Wright et al., 1998). Other studies have commonly recruited via sexual health clinics and/or drug services (Maxwell et al., 2019). However, this was ruled out as potentially biasing the study towards people engaging in medicalised forms of support. Therefore, participants were primarily recruited via the hook‐up/dating app Grindr, a geolocated app that shows other users in relation to oneself, it is also used to organise chemsex hookups. A profile was set up with a neutral picture of the researcher stating that they were interested in talking with people about their experiences of chemsex. No direct approaches were made, only responses to expressions of interest. GPS location was “spoofed” (set to different locations) around Greater Manchester, UK and left to run at a variety of times. Conducting the research in this locale was particularly important as previous research has almost exclusively been conducted in larger capital cities, where the prevailing economic conditions have been highlighted as contributing to the changing dynamics of chemsex (Hakim, 2019). Thirty‐five participants engaged with the researcher and were sent information sheets, 17 were subsequently recruited. Three participants were recruited via snowball sampling.

Several participants enquired about confidentiality, they were reassured it was paramount and would only be broken in compelling circumstances. No disclosures were necessary. In addition, because the chemsex scene in Greater Manchester is relatively small, it was negotiated that minor details would be changed to ensure confidentiality.

To facilitate disclosure, interviews were conducted wherever participants felt comfortable (Wright et al., 1998) including university premises, participants' homes and gardens, coffee shops and pubs. Interviews averaged 118 min ranging from 56 to 173. First interviews followed a semi‐structured guide covering: general background and demographic information; definitions of chemsex and experiences; sexual health; mental health; media and film representations; closing questions; and, participant debrief. The debrief was conducted after the audio‐recording was stopped. Participants were asked how they were feeling, and any support needs they may have had were explored to ensure follow‐up help was offered where needed. Text messages were sent 1 or 2 days after each interview thanking participants for their involvement and to ensure their welfare. Participants were free to contact the researcher informally for the duration of the project. In all but one case no additional referrals were needed to support services. Interviews were conversation‐like to encourage mutual sharing of experiences and to build rapport (Birch & Miller, 2000). Participants were requested to participate in four interviews over 2 years: nine participants completed four interviews; two participants completed three interviews; one participant completed two interviews; and seven participants completed one interview. Reasons for attrition were: moved abroad (*n* = 1), too busy (*n* = 1), said everything they feel they have to say (*n* = 2), unknown (*n* = 7). Interviews were conducted between April 2017 and July 2019.

All participants were over 18 and had recent experience of chemsex. Nineteen of them identified as gay, one identified as bisexual, all were cis‐gendered men. They ranged in ages from 20 through to 58 years old at the start of the study, and were from a range of ethnic and socioeconomic backgrounds.

### 2.1 Analysis

Interviews were coded using NVivo 12, as themes emerged research questions were developed and analytical memos written (Charmaz, 2006). Longitudinal interviews enabled sharing of analytical ideas with participants. First, analysis and reflections from individual participant's own interviews would be shared in subsequent interviews to explore how and why their perspectives developed. Secondly, broader analysis emerging from across other participants were shared. Such processes ensured participant checking of the analysis which helps to enhance rigour (Goldblatt et al., 2011).

#### 2.1.1 Ethics

Ethical approval was granted by The University of Manchester ethical review committee reference 2017‐0820‐2376.

## 3 FINDINGS

The data presented pertains to one theme from the interviews, namely support, care and peer support. It extends to nine of the 20 participants: two had minimal support needs, four had moderate and three had extensive. This confirms the findings by Platteau et al. (2020) that chemsex is not a binary of problematic vs hedonistic practice, and that experiences vary depending on contextual factors. It also demonstrates that for the majority of participants chemsex formed an unproblematic part of their life which needed little or no support. The sub‐themes within this topic are: chemsex services; varieties of peer support; and, the utility and limits of peer support.

### 3.1 Chemsex services

As is commonplace across the UK, chemsex services are based in sexual health clinics, not drugs services. In keeping with this, participants in the study who accessed professional support did so via sexual health clinics. However, these clinics often showed little interest in their health beyond biomedical data gathered via blood tests and swabs. See for example John's comments:

…it was very medical. I never felt that my head and my spirit were ever considered in anything… It was very much these are your bloods

The “your bloods” refer to his HIV follow‐up care. John is a 39‐year‐old black man working in the public sector in various support roles. Since his diagnosis he has been stable on his treatment and has remained undetectable, his treatment was a success. However, the clinic failed to address his “head and spirit” which John latterly described in the interview as being very problematic. Up until his HIV diagnosis he had rarely used any drugs, and had only ever used them in the context of clubbing, not sex. He recounts how upon being diagnosed as HIV positive he “hit the fuck it button” engaging in chemsex frequently for over a year, never to the point of causing himself or others harm, but to the point of causing concern to his friends. Yet because his bloods were fine no further questions were raised.

Mark provides an example of where care did (eventually) come to include something more than bloods. He is a 34‐year‐old white British man who works long hours in retail. His drug use has at times led to him being unemployed. Whilst he moved away from Manchester after the first interview, he visited regularly for his HIV care, so we synced interviews up with his clinic appointments. He takes his HIV medications regularly and his treatment was also considered by medical staff to be a success, but unlike John his drug use was not motivated or exacerbated by his HIV diagnosis. However, at the clinic he noted he was not receiving timely support:

I was turning up to appointments, when I wasn't sober, it was obvious… I was slurring my words, because I'd been taking too much G[BL]… I ask for help in very bizarre ways. I just tell people more and more what I think is going on, in the hope that they're going to turn around and say, do you need something? …the only time they helped me, was when… I started shaking through an appointment, because I had no G[BL], and I couldn't afford to get any, and I was starting to enter withdrawal. That's the only time they really helped me. They admitted me… If somebody had sat down with me properly, talked it through with me… I was more than willing to accept help, I was just not sure how to go about getting it.

Mark was very clear that he was presenting as someone with problematic drug use which escalated to a dangerous state of GBL withdrawal. The first time his use became problematic he was admitted to a medical ward and given diazepam to manage the withdrawal but received no psychological support, nor did he have any peer support network to draw on. He relapsed and he was readmitted after having put himself in danger of serious harm. His second admission was to a specialist mental health ward where he formed connections with other patients who continued to serve as an important source of peer support after his discharge. He has not been readmitted since. He suggested that both admissions could have been prevented with simple questions, such as:

Mark: “How often are you doing it [chemsex]? Is it something more than just happening on a weekend? Start digging into, how often and when it is that they're doing it… Someone might feel as if they're being personal a bit, but if I was asked those questions, I would be quite happy.”

A lack of a support and assessment also extended to patients' sex lives. Ryan is a 49‐year‐old White British male, also HIV positive. Whilst he identified as gay, he had previously been married and had three children. He was open about drug use with them. At the start of our interviews, he took a relatively pragmatic approach to chemsex, acknowledging that it could at times be dissatisfying or problematic, but generally argued, it could enhance pleasure. However, by the third interview 18 months after the first, and after several negative and distressing experiences, his perspective had changed. He had decided to stop chemsex and started attending a local chemsex support group, he noted it had “really helped me with recovery from substances” but that “there wasn't really any support about how to put your sex life back into context”. Similarly James, a younger HIV‐negative participant who had been using drugs for a few years, said “the variety and the type of sex I was having was 100 per cent better when I was on chems”. He noted that he had sex with a wider variety of people and had kinkier and more adventurous sex, something that he valued and experienced as a loss. Likewise, Mark said “The only thing I can't leave behind is the sex… that is what I struggle with”. These participants were therefore left in a difficult position in that as problematic as chemsex had become, it was nonetheless intrinsically tied to their experiences of sexual pleasure, which sobriety significantly diminished. Furthermore, despite being in regular contact with sexual health services, they received little to no support for finding pleasurable ways to have sex outside of a chemsex context. As Mark sums it up “I don't know who I would speak to about it [sex]”. This appears to be at least in part because services prioritised biomedicalised measures and assessments of health, and fail to draw on social networks where sex can be discussed in the context of reduced or eliminated drug use.

### 3.2 Varieties and utility of peer support

Some participants accessed organised group support consisting exclusively of gay and bisexual men. Jack is 51 years old and is white British, and proudly working class, he is from Essex but has lived in Manchester 10 years. He summarised the characteristics of the chemsex group as follows:

I was impressed… I explained that I did have some experience with chemsex, told them what my experiences were… I found them very welcoming… very non‐judgmental. Really what I think a support group should be… [there was also] a great deal of humour in there as well, which I quite liked… some things you do when you're off your face are funny, people were laughing because they'd done it themselves. I suppose… it's one of the few times people will relax and laugh without drugs… I came away thinking they are helping each other.

The amount of paid labour and organisation involved in the group was also relatively minimal, as noted by Ryan:

It's good. It's peer support… they [local charity] facilitate the room and they facilitate a little bit of support but it's more about what you offer to each other.

Ryan also noted that it was a way for him to debrief when he experienced psychologically distressing moments:

yesterday, I started thinking about slamming [injecting] and then it… spiralled out of control… I was sweating and I was shaking… and thinking, god, I just want to have a slam now… I drove straight to my meeting [that evening], to my group, and offloaded on them.

Some participants also offered peer support in immediate ways. John, when faced with someone who had taken too much GBL and was unconscious in his bedroom, ensured their safety:

John: “I've always been a bit of a support‐giver… my autopilot was to look after somebody”

Interviewer: “What would have been signs of deterioration for you then?”

John: “If his breathing changed, if he was being sick… If he fell into some kind of really, really deep sleep…”

Responding in part to reports of gay men not being caring for one another and instead forcibly removing people who had overdosed, John suggested that the opposite occurred:

it would have been wrong of me to turf somebody out like that, wouldn't it? It just came from a very natural place for me

Emotional and practical support also extended beyond gay peers to encompass female, and straight male friends. Robert was the oldest participant at 58, the interviews with him were wide‐ranging, and often full of humour. He is white but grew up overseas in various Asian countries, all his natal family live abroad. He has been HIV positive since the mid‐90 s and moved to the UK because HIV treatment was accessible. He rarely spoke about his wider social life but did speak about a “friend of 30 years” who:

knows that I go out and that I do drugs but she doesn't know to what extent… I'll tell her that I'm going into Manchester then she knows that I'm not going to surface until Thursday or Friday. She's developed her own little monitoring system… if I've not been on Facebook for more than 12 hours she'll phone. If I don't answer she'll then knock‐on the door.

Similar examples existed with another participant Tyler, a white British gay male and the youngest participants at 21. He was studying at university and his straight male friends provided important networks of support very similar to Robert's. These friends had little to no insider knowledge and experience of chemsex or gay male sub‐cultures, but were by virtue of being open to the care needs of others were able to offer support.

Finally, the interviews themselves offered a form of peer support for many of the participants. James summarised his experience of being interviewed as follows:

I thought about things that I wouldn't have thought about without having them posed as a question… I wouldn't have thought to ask them to myself or answer them things … you want to understand and over time we've developed a friendship. Yeah, and that's allowed me to open up more

The interviews offered a space for participants to talk openly and explore their experiences of chemsex, rather than regulate them. Similar sentiments were expressed by Nigel a 30‐year‐old lawyer from an Indian background. Our interviews were always conducted over food at his house. In the first interview, he disclosed that he was HIV positive and was fairly certain this happened at a chemsex party, after which he stopped doing all drugs for at about a year. He restarted doing some drugs during the research. He said that:

It's been interesting to see how I've evolved over time. I've changed in my appetite for it… I do feel usually quite drained after these [interviews], because I think I speak quite frankly… I don't leave any topic sort of withheld… I think it's been in essence cathartic… Thanks for giving me a chance to say these things out loud and in confidence as well, in both a sort of critical but yet therapeutic angle.

In summary peer to peer conversations offer significant support and opportunities for reflection, but wider friendship networks can also be beneficial. However, such conversations did not tend to occur between gay men who have chemsex with one another. Instead, all participants noted there was rarely contact with the chemsex community outside of hooking up. It is therefore important to consider how the growth of peer support networks can be developed and assess what support friendship networks may also offer.

### 3.3 Limits of peer support

Whilst important, peer support could also be fragile. Barry was one of the interviewees who only attended one interview. He was a 27 years old white British gay man with a keen love of art and illustration. He brought some of his work to the first interview as it helped him make sense of some of the complexities of chemsex. Barry differed from other participants, whilst almost all participants eschewed the idea of internalised homophobia as being a key factor in their drug use, Barry was clear that it was for him. Many of his problems stemmed from being a young child when his father beat him to the point of hospitalisation, all because he perceived him as gay. He was clear that he extensively used drugs, to the detriment of his physical and mental health, to suppress these complex and distressing memories so that he could then have sex. In part because of the complex relationship he had to his sexuality, he struggled to engage with gay men as a means of support but had one key female friend:

My group of friends has literally narrowed itself down to one person… and then to see the effect it's had on her, that's also too much for me and just leaves me guilt ridden … I've now lived with this girl rent free for three months, and for three months she looked after me… Can you imagine what that's done to her mental state. For me to come out of the house now on my own, I kind of check in all the time just so she doesn't worry. I can't be left on my own. I know I'm fine, but for her sake, she can't worry that I'm going to go missing.

Undoubtedly, the female friend has been a key source of support. However, he has a pressing feeling of guilt on being so reliant on one person, this leaves him vulnerable. Similarly, there was also a complex fragility to how James' support network when his friend began to struggle with his own mental health:

We used to be really close … but this last year his mental health has deteriorated, … We stopped relying on each other because we have our own shit to deal with … There's not really been anybody else…

James was 22 years old, from a working‐class background and struggling at University, not academically but because of severe mental health problems. Around the same time that his friend was experiencing difficulties James was trying to access support via his GP who declined to provide him with anti‐depressants and mood stabilisers, or a referral for further support. Sadly, only after a suicide attempt was a referral made to a psychiatrist who provided both these things, resulting in his health improving. In summary, whilst peer support and friendships were valuable, they were precarious and cannot be considered a substitute for timely professional support.

## 4 DISCUSSION

There remains much to be done to improve assessment and support for gay and bisexual men who experience problems with chemsex. The findings suggest there is little contact between people who engage in chemsex outside of the activity itself. Reasons for this silence are unclear but could include the stigma (Frederick & Perrone, 2014) and taboo nature (Milhet et al., 2019) of chemsex leading to participants maintaining a degree of separation from their broader personal and professional lives. Much of this stigma and the taboo nature of chemsex likely finds its roots in homophobia and prohibitionist strategies to drugs (Stevens & Forrest, 2018). Research into the barriers for peer support amongst people who currently engage in chemsex would be beneficial. Despite potentially difficult socio‐political contexts, peer support is still valuable but there is scant research addressing the value of peer support in chemsex in relation to effectiveness and best practices, both in relation to its day‐to‐day running and broader organisation within healthcare infrastructures. This is important to consider as some non‐chemsex peer support programmes have been criticised for how they merely extend and repeat coercive forms of medical governance, rather than helping to expand the possibilities for care (Fox et al., 2005). Given that chemsex is prone to these impulses of neoliberal population management and pastoral governance, it is important to consider the conceptual frameworks and histories that are beneficial to draw on. We wish to suggest that HIV contains a rich history of grass‐roots campaigns that were pioneered by LGBT+ people in the 1980s in the face of somewhat similar socio‐political indifference and prejudice, and that such campaigns resisted the coercive forms of medical governance that we are critical of above.

HIV‐based peer support proved to be highly beneficial during the AIDS crisis because the different priorities of clinicians and HIV positive gay and bisexual men were more easily recognised in peer‐based settings (Fredericksen et al., 2020). Peer‐based support also argued for aspects such as self‐confidence, self‐esteem and a positive sense of identity to form a core part of care outcomes (Mowbray et al., 1998). In time this influenced the concept of what successful treatment was, leading to the incorporation of these aspects into HIV care (Lazarus et al., 2016) and improving clinical outcomes by providing patients with experiential information and strategies (Peterson et al., 2012). Peer support also offered a safe space for HIV positive gay men to discuss their sex lives (Paxton, 2002).

Little research exists for peer support in relation to chemsex, however the issues that gay and bisexual men are facing in relation to chemsex share common threads with HIV/AIDS: medicalised models of care; clinicians not prioritising emotional and psychological well‐being; difficulties in discussing one's sex life; and poor clinical outcomes. Hence, just as the problems share similarities, so may the solutions: an approach to peer support that both embraces it as providing important perspectives on chemsex, but also broadening out the meaning of health in relation to chemsex.

Peer support is also protective for health. For example, participants with the most fragile support networks such as Barry, James, John and Mark tended to experience the most severe problems with chemsex. Chatzidakis et al. (2020) highlights that giving such care can be rewarding and foster a sense of community and fulfilment, this was certainly the case during the AIDS pandemic. However, they also highlight caring requires access to appropriate support structures that are fostered at the micro level between ones kin, all the way through to the level of the state. Again, HIV/AIDS activists pioneered not just peer support networks, but models of mutual support that ensured comprehensive systems were in place to ensure the care needs of everyone, cared for and carer, were being met (Katoff and Dunn, 1988). Further research on the lessons that can be learned for chemsex would be beneficial.

The literature on peer support is generally clear that peers are those who share a stigma or characteristic (Dennis, 2003), there is some suggestion from the data that friends without experiential knowledge of chemsex may still be able to offer support. This finds agreement with the history of HIV/AIDS care where lesbians were key organisers and providers of care for a disease which rarely impacted them medically, but was provided out of a deep sense of allyship (Brier, 2007). The extent to which this could be implemented in any sort of formally organised support networks again would need to be the subject of further research and potentially healthcare activism.

Finally, there may also be a role for patients and peers to educate service providers about the breadth of chemsex experiences, and support needs beyond medicalised models of care. Again HIV/AIDS care provides examples where the power and inadequacies of medicalised models of care were challenged, and broader support structures advocated for (Brashers et al., 2000; Peterson et al., 2012). Such models would likely benefit from drawing on the emerging literature in the arts and humanities where the socio‐political structures that surround chemsex are highlighted as being contingent on producing understandings of what chemsex is, and what its effects are (Møller & Hakim, 2021). By extension, amelioration of chemsex's complications and enhancement of its pleasures are contingent on the social and political structures that produce or preclude support from friends and peers. Therefore, following the work of Pires et al. (2022) in addition to the care work that friends and peers perform, support should also help critique and develop services, rather than be co‐opted into existing medicalised forms of chemsex governance.

## 5 IMPLICATIONS FOR POLICY AND PRACTICE

Whilst many people in this study needed little to no support from clinicians, for those that did, assessment and provision of support was partial and tardy. There are particular gaps for psychological and sexual needs. Peer support is a helpful strategy for these needs, as is support from friends. However, peer support networks can be fragile, and their support needs must be considered. Additionally, creating meaningful spaces for patients to engage with service providers may help develop non‐medicalised forms of knowledge that can be used to improve service provision.

## 6 STRENGTH AND LIMITATIONS

Support, care and peer support emerged as a key theme with nine of 20 participants. Therefore, whilst there is a richness and depth to the longitudinal nature of the data, the sample size is particularly small. To some extent, this has enabled a broader understanding to emerge as there were no pre‐conceived ideas that directed recruitment to specific services or settings. Future research should aim to maintain this more capacious understanding, whilst exploring this topic in greater detail in a wider range of locations and settings and including interviews with all those who offer support and peer support.

## 7 CONCLUSION

As with other healthcare issues faced by gay and bisexual men, support for chemsex remains medicalised. The care work that friends and peers perform should help critique, rather than be co‐opted into, existing medicalised forms of chemsex support. Evidence presented above suggests peer support can be of benefit to people who experience problems with chemsex, though the socio‐political contexts of gay sex and drug use can stifle its effectiveness. As with HIV support, the notion of “peer” may be more expansive. Finally, peer networks can be fragile, and care must be offered so that they are supported in caring.

## AUTHORS CONTRIBUTIONS

MN performed project management, data collection, data analysis and drafted the paper; SK reviewed pertinent literature and commented on drafts of the paper.

## FUNDING INFORMATION

This research was in part funded by The Sexually Transmitted Healthcare Foundation, and The University of Manchester Student Experience Internship funding programme.

## CONFLICT OF INTEREST

There are no conflicts of interest to declare.

## Data Availability

Due to the nature of this research, participants of this study did not agree for their data to be shared publicly, so supporting data is not available.
